# Plant Pollen Grains: A Move Towards Green Drug and Vaccine Delivery Systems

**DOI:** 10.1007/s40820-021-00654-y

**Published:** 2021-05-15

**Authors:** Siavash Iravani, Rajender S. Varma

**Affiliations:** 1 Faculty of Pharmacy and Pharmaceutical Sciences, Isfahan University of Medical Sciences, Isfahan, Iran; 2 Regional Centre of Advanced Technologies and Materials, Czech Advanced Technology and Research Institute, Palacký University in Olomouc , Šlechtitelů 27, 783 71 Olomouc, Czech Republic

**Keywords:** Pollens, Sporopollenin, Drug delivery, Vaccine delivery, Plant pollen grains, Microcapsule shells

## Abstract

Pollen grains and plant spores have emerged as innovative biomaterials for various applications such as drug/vaccine delivery, catalyst support, and the removal of heavy metals. The natural microcapsules comprising spore shells and pollen grain are designed for protecting the genetic materials of plants from exterior impairments. Two layers make up the shell, the outer layer (exine) that comprised largely of sporopollenin, and the inner layer (intine) that built chiefly of cellulose. These microcapsule shells, namely hollow sporopollenin exine capsules have some salient features such as homogeneity in size, non-toxic nature, resilience to both alkalis and acids, and the potential to withstand at elevated temperatures; they have displayed promising potential for the microencapsulation and the controlled drug delivery/release. The important attribute of mucoadhesion to intestinal tissues can prolong the interaction of sporopollenin with the intestinal mucosa directing to an augmented effectiveness of nutraceutical or drug delivery. Here, current trends and prospects related to the application of plant pollen grains for the delivery of vaccines and drugs and vaccine are discussed.

## Highlights

- Plant pollen grains and plant spores have emerged as innovative biomaterials for various applications.
- Current trends and prospects related to the application of plant pollen grains for the delivery of vaccines and drugs are discussed.

## Introduction

**Fig. 1:**
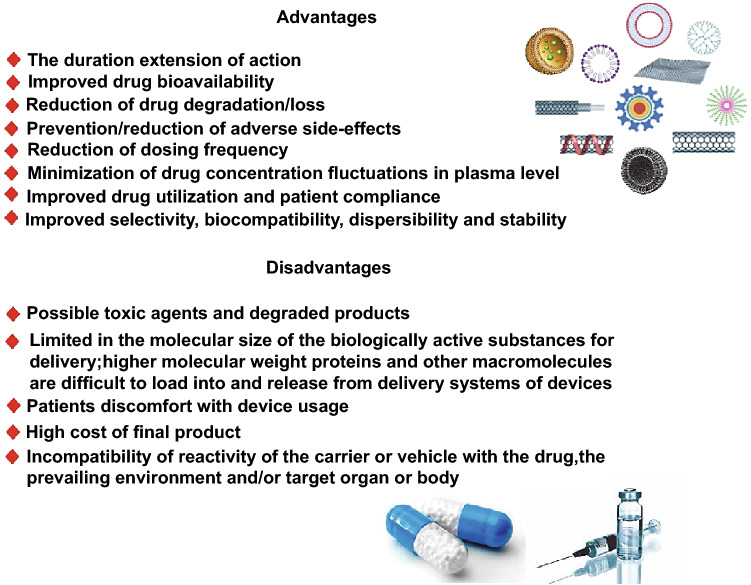
Targeted drug delivery systems/carriers: important advantages and demerits. (Color figure online)

Different techniques have been studied for improving the drug delivery systems to provide high selectivity, specificity, biocompatibility, stability, dispersibility, and controlled release features. The controlled and targeted drug delivery systems typically consist of carrier systems or agents to deliver the drug to the targeted organ and its subsequent release in a programmed manner (Fig. 1) [1–4]. Adhering to the green chemistry values helps to develop eco-friendly drug delivery systems that avoids the utilization of hazardous/toxic elements in the manufacturing procedures and enables lower-dose medicines for the treatment. The applications of materials/ingredients with high biocompatibility and low toxicity in pharmaceutical/medical formulations can reduce/prevent the possible adverse side effects emanating from the pharmaceutical residues entering the body or environment. In this regard, different types of pollen grains are widely distributed with specific/unique sizes and morphologies as well as good biocompatibility [5–9]. However, among these diverse types of pollen grains, almost all documented work predominantly relates to *Lycopodium clavatum* and *Populus deltoids* species because of their availability from standard chemical product suppliers [10]. Hollow sporopollenin shell from spores or pollen can be obtained via the removal of proteins, cytoplasmic materials, and the intine layer (which is made of cellulose and pectin) underneath the exine layer without damaging the structure [11]. Sporopollenin is composed of oxygen, hydrogen, and carbon (C_90_H_144_O_27_) and contains methyl and hydroxyl groups with a regular and uniform shape and size distribution, large internal cavities and interconnected pores, being suitable for drug encapsulation [12]. The shells are biocompatible and resistant to harsh chemicals conditions, including organic solvents, acids, and alkali. Additionally, they have good thermal stability and are an abundant and sustainable natural source [11, 13]. Notably, the materials inside the pollen shell comprise various proteins, which can make allergic reactions, thus it is crucial to eliminate the cytoplasmic content of the pollen before their biomedical and clinical applications. Though, the chemically processed protein-free pollen is not always neutral toward the immune system, as has been indicated that protein-free ragweed pollen could interact with dendritic, intestinal epithelial cells and macrophages, resulting in the release of inflammatory cytokines and chemokines [5, 14–17]. The immunomodulatory potentials of ragweed pollen can be deployed in effective delivery of drugs, but more elaborative studies should be undertaken for the biomedical applications of these pollens [18]. Owing to their unique properties, sporopollenin shells can be considered as suitable candidates for the encapsulation and delivery of various polar and nonpolar drugs [19–22].

There are various chemical and enzymatic techniques for extracting the shell from either pollen or spore. Generally, various alkali and organic solvents have been utilized to eliminate the cellulosic layer, lipids, and genetic contents of the sample [23–25]. Using chemical methods, the structure can be separated after removing the polysaccharide intine via treatment with diluted acidic solutions [13, 26]. For pharmaceutical and biomedical applications, several investigations have focused on replacing toxic acid/alkali regents with eco-friendly reagents, including bio-based ionic liquids with their unique solvent properties that can dissolve various biopolymers; however, they are expensive and toxic [27–29]. Thus, different materials, including silica, carbon nanotubes and polystyrene should be explored as the supporting materials for these solvents to obtain the supported ionic liquids [8]. Additionally, sporopollenin can be obtained via enzymatic isolation techniques by exploiting various enzymes such as pectinase, pronase, cellulysin, amylase, lipase, and hemicellulase [30–32].

The inimitable sporopollenin’s physicochemical characteristics provoke the abstraction of sporopollenin exine pods from pollen barriers as a sustainable and renewable resource of organic microcapsules for appliances in encapsulation [11]. In one study, the effect of polymer coating on drug loading and release properties of sporopollenin microcapsules extracted from date palm (*phonix dactylifera L*) were evaluated. Both of the carboxymethyl cellulose/epichlorohydrin-coated and chitosan-coated capsules recorded a maximum drug loading of 97.2% with 50 mg mL^−1^ at pH 6.0–6.4. The faster release was revealed when the pH increased from 1.4 to 7.4 in both the coated capsule samples [19, 20]. The release of drugs from the loaded sporopollenin shells was limited at low (1.4) and high pH (> 6). It was disclosed that this slow release could be due to repulsion forces on the adsorption sites between either H^+^ or OH, and the examined paracetamol at low and high pH, respectively. The release behaviour from the shell can broadly be influenced by the polymer employed for coating of the shell which should be considered separately when sporopollenin is utilized for drug release investigations [14, 20]. Remarkably, the electrostatic repulsion forces and acidic/basic conditions of the media have some effects on the solublity of the drug, and they can also affect the loading/release behaviour from the shell [19, 20]; the solvent media do control the release of active substances [13]. In this review, recent advances related to the application of plant pollen grains for the delivery of drug/vaccine are highlighted.

## Drug Delivery Applications

Before the application of pollens for biomedical and drug delivery purposes, their inherent biomolecules occupying most of the inner cavity of pollen should be eliminated not only to create void room, as their presence may also initiate allergies upon in vivo administration [33]. The materials present in the pollen interior need to be extracted via chemical means to prepare pristine pollen skeletons. Typical methods include a series of sequential treatments with organic solvents, alkalis, and acids to eliminate the native pollen biomolecules. For instance, pristine pollen shells can be generated from assorted plant species deploying typical chemical processing [33] wherein technique successively deployed acetone, phosphoric acid, and hydroxides; ensuing shells have been successfully produced with clean and intact hollow structures from various pollen species such as ragweed, sunflower, black alder, and lamb's quarters [33].

The application of various naturally abundant, nontoxic pollen grains was illustrated for producing platinum‐pollen hybrid microrobots with the potential appliances in biomedicine field [34]. Assorted pollen grains were employed originating from pine, dandelion, lotus, camellia, sunflower, poppy, cattail, galla and lycopodium that exhibit the sturdiness of various kinds of pollen grains as drug carriers. Accordingly, the designed microrobots had enough safety aspects which expand their potential application in biomedicine and drug loading [34]. For increasing the filling capacity and long-term absorption, plant exine capsules (natural pollen grains) have been employed with large internal cavities for loading and robust exine against harsh conditions [35]. Admixed solution forms of glycerol monostearate and nobiletin were prepared in the plant exine capsule’s internal cavities via ultrasound at elevated temperature to fabricate nobiletin in a supersaturated status, and the ensuing filled pods were cooled to ambient warmth. Under simulated intestinal and gastric settings, alginate-based hydrogels were next chosen for capturing and further regulating the discharge of nobiletin. Accordingly, significant nobiletin loading capacity of 770 ± 40 mg g^−1^ could be attained by using sunflower pollen grains. Importantly, the presence of glycerol monostearate, sunflower pollen grains and alginate-based hydrogels slowed down the synergistic discharge of nobiletin, thereby affording a gradual discharge effect in stomach whereas achieving a long-term effectual assimilation in the intestine [35].

**Fig. 2:**
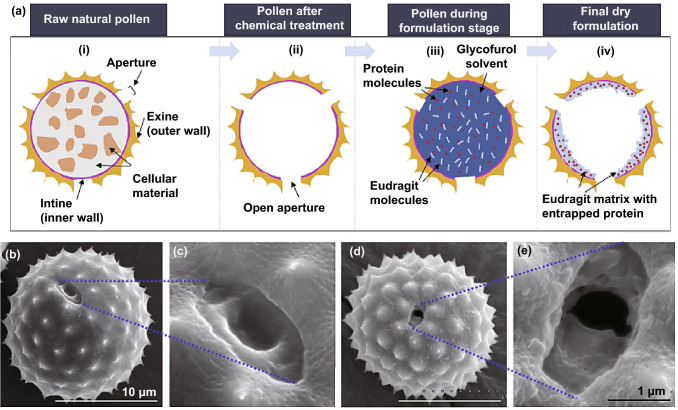
**a** Pollen grains for oral delivery of proteins. Pollen grain-based formulation with scanning electron microscopy (SEM) of raw pollen grain with closed aperture (**b**, **c**), and processed pollen grain with open aperture (**d**, **e**). Reproduced with permission from Ref. [24]. (Color figure online)

Protein-based nanoparticles with suitable absorptivity and low toxicity still experience a major challenge for rapid nutraceutical or drug release after oral administration [25]. In one study, a secondary encapsulation technique was introduced for the controlled release of drugs in gastrointestinal (GI) environment [25]. Accordingly, the assembled nanoparticles engineered by nobiletin, zein, and tannin acid were introduced for the drug delivery systems. The added tannin acid had potentials to produce further assembly of stabilized nobiletin when compared to nobiletin-loaded zein NPs alone. The carriers in the form of sunflower pollens have been deployed for oral administration, whereas zein was selected as a covering substance for capping sunflower pollens grains. The prepared system had a stable size of 100 nm after 48 h. Additionally, the suggested system could enhance the chemical consistency of nobiletin for no less than 120 days at 4 °C when matched with zein NPs. Owing to the secondary capping accorded by sunflower pollens grains, the ultimate system could selectively discharge through oral administration, providing no release in a gastric environment and slow release in an intestine environment [25]. Interestingly, pollen grains from ragweed (*Ambrosia elatior*) were obtained to serve as shields for microcapsules (Fig. 2) [24]. A matrix containing an enteric polymer, Eudragit L100-55, was placed on the interior facades of ragweed pollens to safeguard the encapsulated protein from gastric decomposition and to acquire discharge in the intestine in a pH-dependent manner. The matrix comprising Eudragit L100-55 was prepared in the absence of organic solvents, thus precluding the solvent-induced impairment of protein molecules could be prohibited. Accordingly, a bovine serum albumin-loaded matrix of Eudragit L100-55 was produced in ragweed pollens and its release evaluations in mimicked gastric fluid at pH 1.2 exhibited negligible albumin discharge from the ragweed-Eudragit L100-55 formulations. The assessment of albumin maintained in the formulation subsequent to its gastric fluid exposure revealed that the enduring albumin retained its integrity. The analyses of discharge in the mimicked intestinal fluid at pH 6.8 demonstrated that ragweed pollen provided further regulated discharge mechanism inside the initial few hours of discharge because of their solid wall [24].

**Fig. 3:**
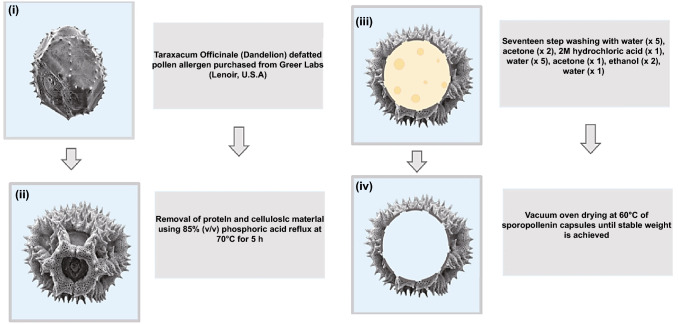
Extraction procedures of cage-like sporopollenin exine capsules from dandelion pollen grains. Reproduced with permission from Ref. [36] (CC BY 4.0). (Color figure online)

**Fig. 4:**
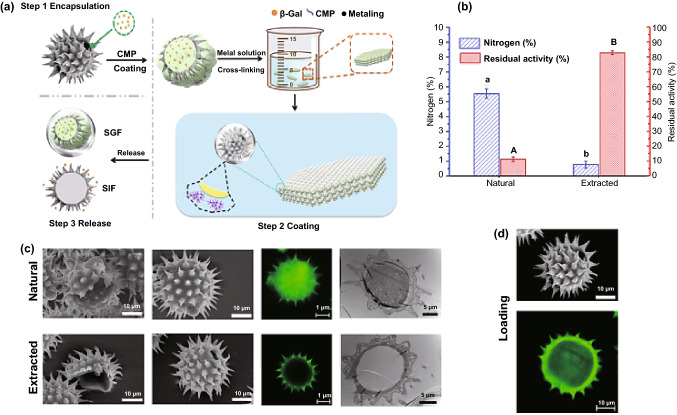
**a**–**d** Design procedures of intestinal protein oral delivery system using pollen. Sporopollenin exine capsules: SECs. Reproduced with permission from Ref. [37] Copyright© 2020 American Chemical Society. (Color figure online)

The extraction and macromolecular loading of dandelion hollow sporopollenin exine capsules have been illustrated [36]. Among the examined procedures, acidic hydrolysis deploying phosphoric acid 85% (v/v) refluxed at 70 °C for five hours afforded an ideal balance of undamaged yield of particle, preservation of cage-like microstructure and protein elimination [36]. For packing purposes, bovine serum albumin has been encased inside the dandelion hollow sporopollenin exine capsules with high efficiency (32.23 ± 0.33%). It was revealed that highly monodispersed, intact and clean dandelion sporopollenin exine capsules could be produced via acidolysis using phosphoric acid at an elevated temperature (Fig. 3) [36]. Besides, an oral distribution medium comprising carboxymethylpachymaran (CMP)/metal ion alteration and sporopollenin exine capsules was engineered with aimed discharge centred on food-grade ingredients and handling procedures (Fig. 4) [37]. As a result, the prepared CMP/3% AlCl_3_ system demonstrated the remarkable capability of controlling the release with the maximum residual activity of *β*-galactosidase (as a model protein) at nearly 72% subsequent to treatment for 24 h. Interestingly, the conditions at low pH were conducive to additional cross-linking of CMP and metal ions, producing a gel of compact assembly and high strength, which could impact the controlled discharge of β-galactosidase in gastrointestinal tract [37].

Paracetamol was loaded into the sporopollenin microcages obtained from the pollens of *Platanus orientalis*, wherein microcages comprising sporopollenin were highly reticulated, physically secure, and thermally durable [38]. The loading efficiency of the sporopollenin microcages was reported about 8.2% by applying the passive filling approach and 23.7% through evaporating packing method. The kinetics evaluations and in vitro discharge were accomplished to evaluate the appropriateness of sporopollenin microcages for packing; such sporopollenin microcages could be deployed for controlled drug delivery applications [38]. In one study, sporopollenin obtained from pollen grains of *Cedrus libani* and *Pinus nigra* was utilized for the delivery of anticancer drug oxaliplatin where its slow release from sporopollenin was demonstrated (~ 40–45 h) [39]. The MYC and FOXO-3 gene expression remarkably augmented in CaCo_2_ cell and reduced among non-cancerous Vero cell affirming that sporopollenin-facilitated regulated discharge of oxaliplatin, which could stimulate the apoptosis cell demise and avoid the dispersion of its adverse influences to neighbouring healthy cells [39]. Additionally, sporopollenin macroporous capsules isolated from date palm (*Phoenix dactylifera* L.) spores and coated by a natural polymer composite (chitosan with glutaraldehyde) were employed in the in vitro-controlled delivery of ibuprofen [20]. According to the Langmuir adsorption isotherm, ibuprofen charging was enhanced when its concentration was decreased; maximum filling of the drug being detected at pH 6.0 (50 mg mL^−1^, 97.2%). The discharging analyses demonstrated that ibuprofen was dispensed faster as the pH was altered from 1.4 to 7.4. Additionally, the cytotoxicity evaluation of the prepared systems against human intestinal Caco-2 cell line displayed good biocompatibility using 3-[4,5-dimethylthiazol-2-yl]-2,5-diphenyl tetrazolium bromide (MTT) assay [20].

Sporopollenin microcapsules isolated from *Betula pendula* pollens were employed for the delivery of cancer therapeutic agent (imatinib mesylate); the encapsulation efficiency by passive loading method was about 21.46% [40]. Additionally, the drug release from microcapsules was noticed to be biphasic, an early release being faster trailed by a gradual rate of discharge. Notably, the discharge of the drug, imatinib mesylate, itself (control) was quicker as compared to sporopollenin microcapsule loaded with imatinib mesylate; the discharge pattern for both, the free and the encapsulated drugs was really gradual and additionally regulated in phosphate-buffered saline (PBS) buffer at pH 7.4 compared to HCl at pH 1.2. Sporopollenin microcapsules entrapped imatinib mesylate’s accumulative drug discharge in 24 h for PBS was found to be 65%, although discharge from the control was finished in an hour. The drug-filled microcapsules have been found to be effectual for human colon carcinoma cell line via MTT assay [40]. In another study, the sporopollenin isolated from *Lycopodium clavatum* spores was utilized for the encapsulation of erythromycin and bacitracin antibiotics [41]; the entrapment and filling competence of erythromycin were 32.4% and 16.2, respectively. The activities of antibiotic-loaded sporopollenin, pure antibiotics, and unfilled sporopollenin have been evaluated against *Pseudomonas aeruginosa*, *Staphylococcus aureus*, and *Klebsiella pneumoniae*. Interestingly, a significant increase in the antibacterial activity was discerned for drug-loaded sporopollenin system, compared to the examined pure antibiotics. The cytotoxicity analyses exhibited that these systems were harmless versus Caco-2, the human epithelial colorectal adenocarcinoma cells. A deviation from Fick's law was illustrated by the in vitro discharge mechanism for erythromycin at pH 7.4. *I* The discharge of erythromycin in vivo from sporopollenin (oral dosage 50 mg kg^−1^) showed remarkable values displaying the improved bioavailability of erythromycin [41].

Naturally occurring and inexpensive sporopollenin exine capsules, derived from the spores of the plant *Lycopodium clavatum,* were employed for the safeguard against light and separation of the bioactive antibiotic, marinomycin A which is light-sensitive [42]; the sporopollenin exine capsules entrapment significantly increased the half-life of the macrodiolide’s exposure to UV irradiation. Especially, they have the short half-life of marinomycins in normal light, which harshly influences their imminent biological effectiveness as they exhibit powerful anticancer and antibiotic action. Additionally, the sporopollenin exine capsules can be employed to selectively extract marinomycins from the culture broths, which offers a remarkably superior retrieval relative to conventional resins while providing concurrent safeguard against light [42]. Besides, sporopollenin exine capsules obtained from spores of the common club moss *L. clavatum* were employed for the protection of ω-3 oil from enhanced oxidation by UV irradiation or oxidation instigated by normal light [43]; the action mechanism was proposed to be mainly governed by free radical quenching rather than to light protection. No material change in terms of antioxidant activity was observed by the abstraction process from the raw material and was evidently an innate attribute of the sporopollenin contained comprising the spores of *L. clavatum*, because of the abundantly available phenolic functionalities on the exterior of these pods [43].

**Fig. 5:**
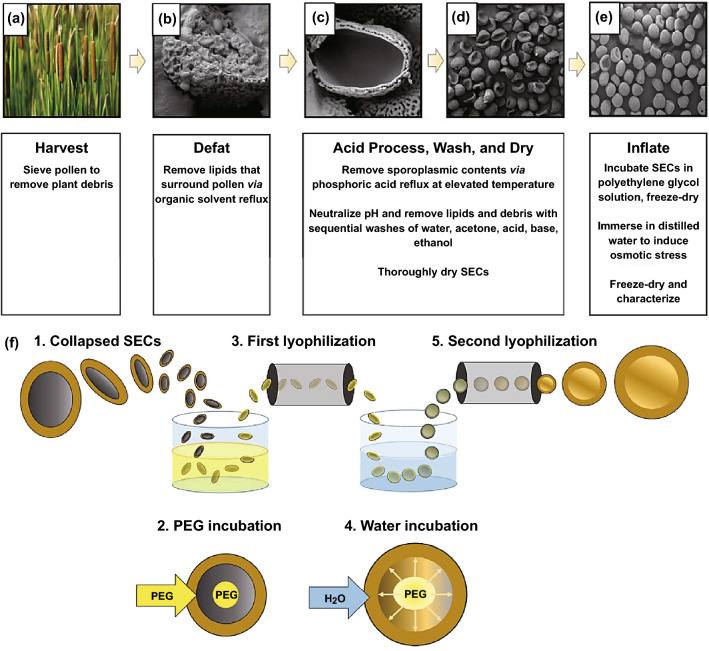
**a–f** Sporopollenin exine capsules (SECs) extraction procedures from cattail (*Tyhphae angustfolia*) pollen grains. PEG: polyethylene glycol. Reproduced with permission from Ref. [44]. (Color figure online)

It is demanding proposition to isolate completely operative sporopollenin exine capsules from various species of pollen, as frequent collapsing of pollen grains incite the lose of structural integrity, bulk consistency and packing volume [44]. In one study, polyethylene glycol osmolyte solutions were utilized to preserve the native architectural properties of the isolated capsules, yielding inflated microcapsules of high uniformity that persist even after subsequent lyophilization. While acid-processed sporopollenin exine capsules suffered extreme levels of structural failure, gestation in solutions of 2.5% or higher polyethylene glycol (PEG) remarkably enhanced the conservation of spherical capsule form by stimulating inflation inside the micropods (Fig. 5) [44].

**Fig. 6:**
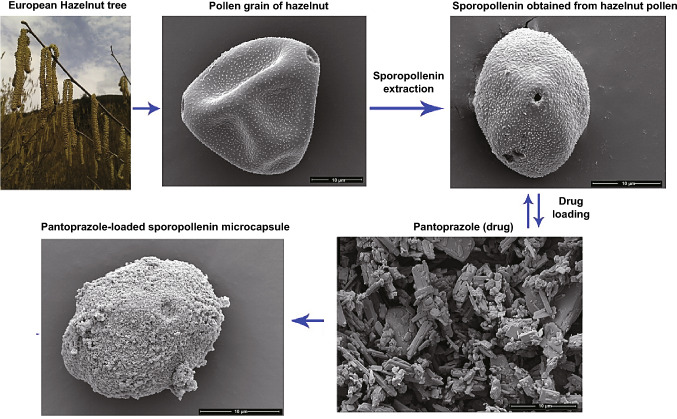
Production procedures of pollen-derived microcarriers for pantoprazole delivery. Reproduced with permission from Ref. [45]. (Color figure online)

Sporopollenin microcapsules were obtained from the pollens of a common tree (*Corylus avellana*) and utilized as a microcarrier for pantoprazole with encapsulation efficiency for the drug being 29.81% [45]. Results from thermogravimetric analyses showed that thermal stability of pantoprazole was improved by encapsulation; in vitro release evaluations revealed that drug-loaded sporopollenin microcapsules had better discharge functions than the control (Fig. 6) [45].

## Oral Vaccination

**Fig. 7:**
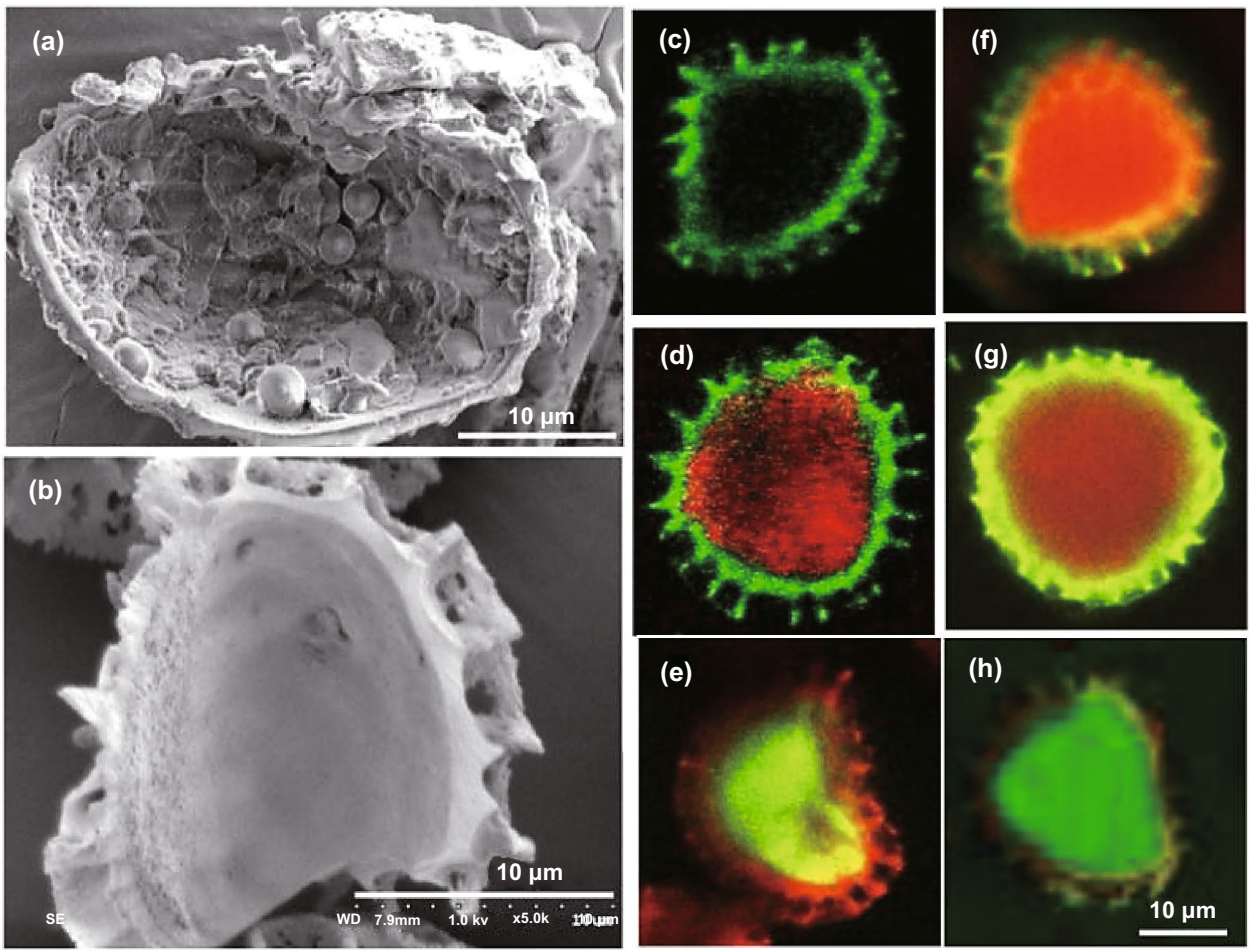
SEM images of lycopodium spores manually cracked (**a**) biomolecules and cellular organelle are observed in the core before processing, and (**b**) a clear core can be observed after chemical processing. The chemical processing of lycopodium spores with their confocal images, empty **(c)**, loaded with sulforhodamine (**d)**, loaded with dextran conjugated to fluorescein isothiocyanate (**e)**, loaded with ovalbumin conjugated to texas red (**f**), loaded with bovine serum albumin conjugated to texas red (**g**), and loaded with dextran conjugated to fluorescein isothiocyanate (**h**). Reproduced with permission from Ref. [46]. (Color figure online)

Oral vaccination can provide effortless and convenient approach to vaccination thereby instigating systemic immunity with promising potential to stimulate mucosal immunity via antigen-processing by the gut-associated lymphoid tissues [46]. As an example, pollen grains were engineered to be employed as simple modular systems for oral vaccination (Fig. 7). It was revealed that spores of *Lycopodium clavatum* could be cleaned chemically to eliminate built-in proteins to produce whole neat empty shells [46]. Consequently, these empty pollen pods could be efficaciously packed with varying sizes of molecules with great potential to be widely deployed as a vaccination arrangement. As a model antigen, spores of *Lycopodium clavatum* formulated with ovalbumin were orally fed to mice where they could stimulate remarkably high anti-ovalbumin fecal IgA antibodies and serum IgG relative to stimulation attained by application of a positive-control adjuvant, cholera toxin; antibody reaction was not influenced by the stomach acid and continued for seven months [46].

**Fig. 8:**
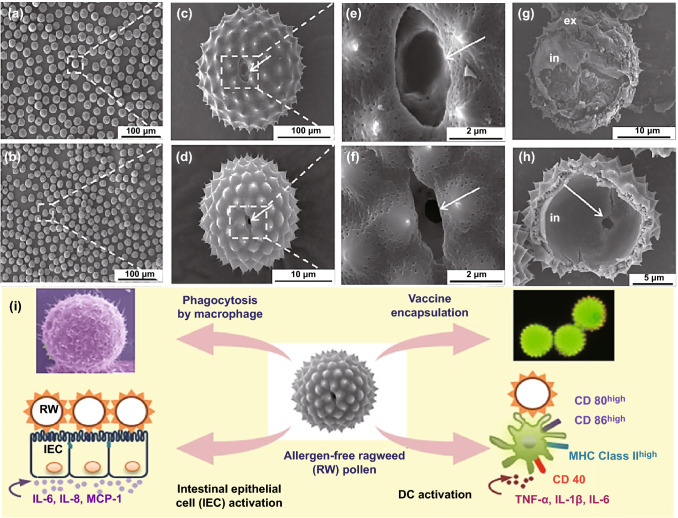
SEM images of ragweed pollen grains before **a**, **c**, **e**, **g** and after **b**, **d**, **f**, **h** chemical treatment. **i** The design of oral vaccine delivery system using ragweed pollens. Reproduced with permission from Ref. [18]. (Color figure online)

Pollen grains have been employed for the delivery of oral vaccines [18]. By applying extensive chemical processing, allergen-free pollen microcapsules were equipped to be loaded with vaccine antigens. The effects of chemically processed ragweed pollen (*Ambrosia elatior*) on the innate immune system have been evaluated (Fig. 8). Consequently, it was revealed that in response to ragweed pollen, intestinal epithelial cells, macrophages, and dendritic cells discharge inflammatory chemokines and cytokines; SEM imaging revealed that macrophages could swamp ragweed pollen [18]. Additionally, mouse dendritic cells upregulated their stimulation indicators, namely CD86, CD 80, CD40, and MHC class II molecules in the presence of ragweed. Interestingly, IL-8 and MCP-1 expression was reduced at higher pollen concentration (4 mg mL^−1^). The ragweed pollens did not inflict cell membrane damages when matched to comparable-sized poly (lactic-co-glycolic acid) particles nor did they influence the epithelial cells in intestine; they could be found in the subepithelial region of the small intestine 24 h after pollens were delivered to mice [18].

Aimed for oral vaccination, Gill et al. [9] evaluated ragweed pollen (obtained from *Ambrosia elatior*) where chemically treated, allergen-free ragweed pollens were produced. Oral dosages (8 weekly) of ovalbumin devised with treated ragweed generated intense systemic (anti-ovalbumin IgA, IgG1, IgG, and IgG2a) and mucosal (anti-ovalbumin IgA) immune reactions, which after vaccination remained for at least 3 months; mucosal IgA versus ovalbumin was reported in the vaginal secretion, saliva, feces, and lung lavage. It should be noted that some evidences show that pollens may have safety issues for oral administrations, but more elaborative and controlled human studies are needed to document their safety. These analyses can then lay the foundation for analysing pollen grain-based oral vaccine formulations in humans with the ultimate objective of developing edible vaccines [47].

## Conclusion and Future Outlooks

Plant pollen grains have shown promising biomedical potentials with their three-dimensional (3D) structures and unique morphologies; they are easily obtainable in larger quantities from abundant and renewable plant sources in an array of shapes and sizes via cost-effective means and simple preparative protocols. These characteristics coupled with their reliability that is assured by identifiable species of origin are some of the salient advantageous features. In pollen grains, the genetic matter is confined by a double-incrusted barrier, which is made up of intine and exine. The former is composed predominantly of pectin, hemicellulose and cellulose, while the latter, termed as sporopollenin is mainly comprised of a uniquely-structured biopolymer that is made up of exclusively of hydrogen, oxygen and carbon atoms.

Sporopollenin microcapsules obtained from various pollen species have been employed as greener drug carriers, because of their good biocompatibility, low toxicity, homogeneity in size, resistance to harsh chemical conditions and high thermal stability. These microcapsules are of particular interest based on their complex architecture, significant strength/elasticity and large internal cavities. Additionally, they are resistant to chemical dissolution and disintegration and at the same time promptly agreeable for modification, because of the existence of an array of functionalities, namely alcoholic, ether, carboxyl, and carbonyl groups. To produce sporopollenin exine capsules for the drug delivery and other biomedical applications, it is very important to develop simple and non-toxic methodology to isolate intact and clean capsules with no evidences of damages on their intrinsic architectures. Notably, some critical factors such as solubility, pressure on the microcapsule, and pH can affect the release behaviour of materials from hollow microcapsules, thus the maintenance of their structural integrity should be systematically and analytically evaluated. Active release can be fine-tuned by applying appropriate coating processes on the shells, or co-encapsulation with the active materials inside the shells. Additionally, pollen grains can be chemically processed for the modification of their structural features while preserving their valuable innate microscale features. This article hopefully can stimulate further investigations embracing the aforementioned strategies.
